# Management and outcome of cutaneous diphtheria in adolescent refugees in Germany, June 2022 – October 2023

**DOI:** 10.1007/s15010-024-02374-y

**Published:** 2024-08-27

**Authors:** Alicia Zink, Juliana Hofer, Christian Schneider, Franziska Kessler, Hannes Klenze, Dietrich Klauwer, Klaudia Maleki, Andreas Müller, Sarah Goretzki, Shubei Wang, Robin Kobbe, Andrea Vanegas Ramirez, Sebastian Bode, Ales Janda, Roland Fressle, Jonathan Remppis, Philipp Henneke, Siegbert Rieg, Anja Berger, Andreas Sing, Markus Hufnagel, Benedikt D. Spielberger

**Affiliations:** 1 Division for Paediatric Infectious Diseases and Rheumatology, Department of Paediatrics and Adolescent Medicine, University Medical Centre, Medical Faculty, University of Freiburg, Freiburg, Germany; 2 Institute for Medical Microbiology and Hygiene, University Medical Centre Freiburg, Freiburg, Germany; 3 Centre for Child and Adolescent Health, Hegau-Bodensee Clinic Singen, Constance District Health Association, Singen, Germany; 4 Department of General Medicine and Health Services Research, Heidelberg University Hospital, Interdisciplinary Refugee Outpatient Clinic at the Patrick-Henry-Village State Initial Reception Centre, Heidelberg, Germany; 5 Initial Reception Centre of the State of Hesse, Giessen Regional Council, Giessen, Germany; 6 Clinic for Paediatrics I, Essen University Hospital, Essen, Germany; 7 Pediatrics 2: General and Special Pediatrics including Pediatric Infectious Diseases, Klinikum Stuttgart, Olgahospital, Stuttgart, Germany; 8 Institute for Infection Research and Vaccine Development, University Medical Centre Hamburg- Eppendorf, Hamburg, Germany; 9 Department of Infection Epidemiology, Bernhard Nocht Institute for Tropical Medicine (BNITM), Hamburg, Germany; 10 Bundeswehr Hospital Hamburg, Tropical Dermatology at the Bernhard Nocht Institute for Tropical Medicine, Hamburg, Germany; 11 Department of Paediatrics and Adolescent Medicine, Ulm University Medical Centre, Ulm University, Ulm, Germany; 12 Paediatric and Adolescent Practice Dr Roland Fressle, Freiburg, Germany; 13 Department of Neuropaediatrics, General Paediatrics, Diabetology, Endocrinology, Social Paediatrics, University Children’s Hospital Tübingen, Tübingen, Germany; 14 Institute of Tropical Medicine, Travel Medicine and Human Parasitology, University Hospital Tübingen, Tübingen, Germany; 15 Institute for Infection Prevention and Control, Medical Centre - University of Freiburg, Faculty of Medicine, University of Freiburg, Freiburg, Germany; 16 Department of Medicine II, Division of Infectious Diseases, Medical Centre-University of Freiburg, Faculty of Medicine, Freiburg, Germany; 17 Consultant Laboratory for Diphtheria, WHO Collaborating Centre for Diphtheria, Bavarian Health and Food Safety Authority, Oberschleissheim, Germany; 18 Department of Paediatrics and Adolescent Medicine, University Medical Centre, Medical Faculty, University of Freiburg, Mathildenstr. 1, 79106 Freiburg, Germany

**Keywords:** Diphtheria, Throat diphtheria, Cutaneous diphtheria, Toxigenic *C. diphtheriae*, Minor refugee, Balkan route

## Abstract

**Objectives:**

From September 2022 an increase in *Corynebacterium diphtheriae* (*C. diphtheriae*) infections was reported in Europe. Our study focuses on 31 adolescent and young adult refugees with cutaneous *C. diphtheriae* infections detected in Germany. We examined treatment regimens and outcomes to provide targeted insights into the management of this infection.

**Methods:**

We distributed a standardized survey, focused on children and adolescents presenting to paediatric clinics through the German Paediatric Infectious Diseases Society (DGPI) and additional professional contacts in Germany. Data were extracted from routine medical documentation and reported anonymously.

**Results:**

A total of 31 individuals with cutaneous *C. diphtheriae* infection were reported by 9 centres. Two of these showed diphtheria toxin (DT) related systemic symptoms and four exhibited systemic inflammation requiring complex management. The remaining 25 cases, with exclusively cutaneous manifestations, were afebrile. Treatment with topical antiseptics and systemic antibiotics, mainly aminopenicillin/beta-lactamase inhibitors (BLI) (35%) or clindamycin (25%), achieved eradication in all but two cases treated with aminopenicillin/BLI. Treatment duration varied between 5 and 17 days.

**Conclusions:**

In refugees presenting with chronic skin wounds, *C. diphtheriae* should be included into the differential diagnosis. Fever seems to be a valuable marker to differentiate severe cases with potentially DT-mediated sequelae from exclusively cutaneous diphtheria (CD). For afebrile CD, topical antiseptics and oral antibiotic therapy with clindamycin for 7 days, followed by clinical surveillance appears to be a safe treatment regimen. Patients with CD who present with fever or pharyngitis should be thoroughly investigated including blood and pharyngeal swab cultures.

**Supplementary Information:**

The online version contains supplementary material available at 10.1007/s15010-024-02374-y.

## Introduction

Diphtheria is a uncommon, contagious and potentially lethal upper respiratory tract infection, that can cause systemic illness associated with diphtheria toxin (DT)-related cardiac (e.g., myocarditis, arrhythmia) and neurological (polyneuropathy) sequelae [1–3].

In Western countries – where diphtheria is not endemic –, unspecific wound infections due to toxigenic *Corynebacterium diphtheriae* strains are usually observed in travellers and patients with migration history [4, 5]. From 1st September 2022 until 12th February 2024, 443 cases of *C. diphtheriae* infection were reported to the European Centre for Disease Prevention and Control (ECDC) and most cases were cutaneous infections [6, 7]. Patients with cutaneous diphtheria (CD) often present with one or more erosive skin lesions, primarily localised at the hands and feet, which are prone to wounds and act as entry points for further infection [8]. Co-infection with cosmopolitan infections such as *S. aureus* or *S. pyogenes* was commonly noted [8]. Around 50% of *S. aureus* isolates were methicillin-resistant and resistance against clindamycin and tetracyclines was reported in two studies in up to 10% of the patients [8, 9]. As a response to the emergence of CD in Europe, ECDC issued a rapid risk assessment to foster awareness, and to improve detection, treatment and vaccination against the disease [10]. Additionally, a new guideline on the clinical management of respiratory diphtheria was published by the World Health Organisation (WHO) in February 2024. However, the evidence base for the management recommendations was considered to be weak and guidance on the treatment of CD is lacking [11]. Very recently, microbiological data and outbreak clusters have been analysed and reported [7], but a strategic analysis of the applied treatment regimens, – especially in children and adolescents – is still lacking. We therefore explored treatment regimens as chosen by practitioners and the associated outcomes in individuals suffering from CD, with the aim of developing a practical treatment algorithm.

## Patients and methods

We performed a standardized survey through the German Paediatric Infectious Diseases Society (DGPI) and additional professional contacts in Germany, Switzerland, and Austria. Participating colleagues were asked to provide information on patients with detection of C. *diphtheriae* in skin swabs, who presented to their institutions between 01.06.2022 and 30.09.2023. After sending out two reminders, we have closed the survey on 15.01.2024. The survey targeted children and adolescents up to the age of 22 years to include both younger and older adolescents who still frequently present to paediatric services, particularly in refugee camps. This ensures a comprehensive understanding of how the disease affects this transitional age group. The data was derived from routine medical documentation and reported in an anonymous format. Microbiological susceptibility testing was performed in all centers according to the guidelines from the European Committee on Antimicrobial Susceptibility Testing (EUCAST) for all isolates. Antibiotic resistances were queried for *S. aureus*,* S. pyogenes* and *C. diphtheriae*.

Collected data included demographic data (e.g., date of birth, sex, country of birth), data on escape route, treatment (length of treatment, isolation measures, localization of skin wound(s), topical treatment, systemic treatment including reasons for treatment decision, changes in therapy, duration of therapy and usage of antitoxin), as well as microbiology results (detection of pathogens in wound and throat swabs), detection of diphtheria toxin (DT) including detection method (PCR, Elek test), resistance patterns of detected pathogens and outcome measures. Data was collected and analysed in Microsoft Excel. Given the retrospective design of our study, we focused on descriptive analysis.

**Fig. 1:**
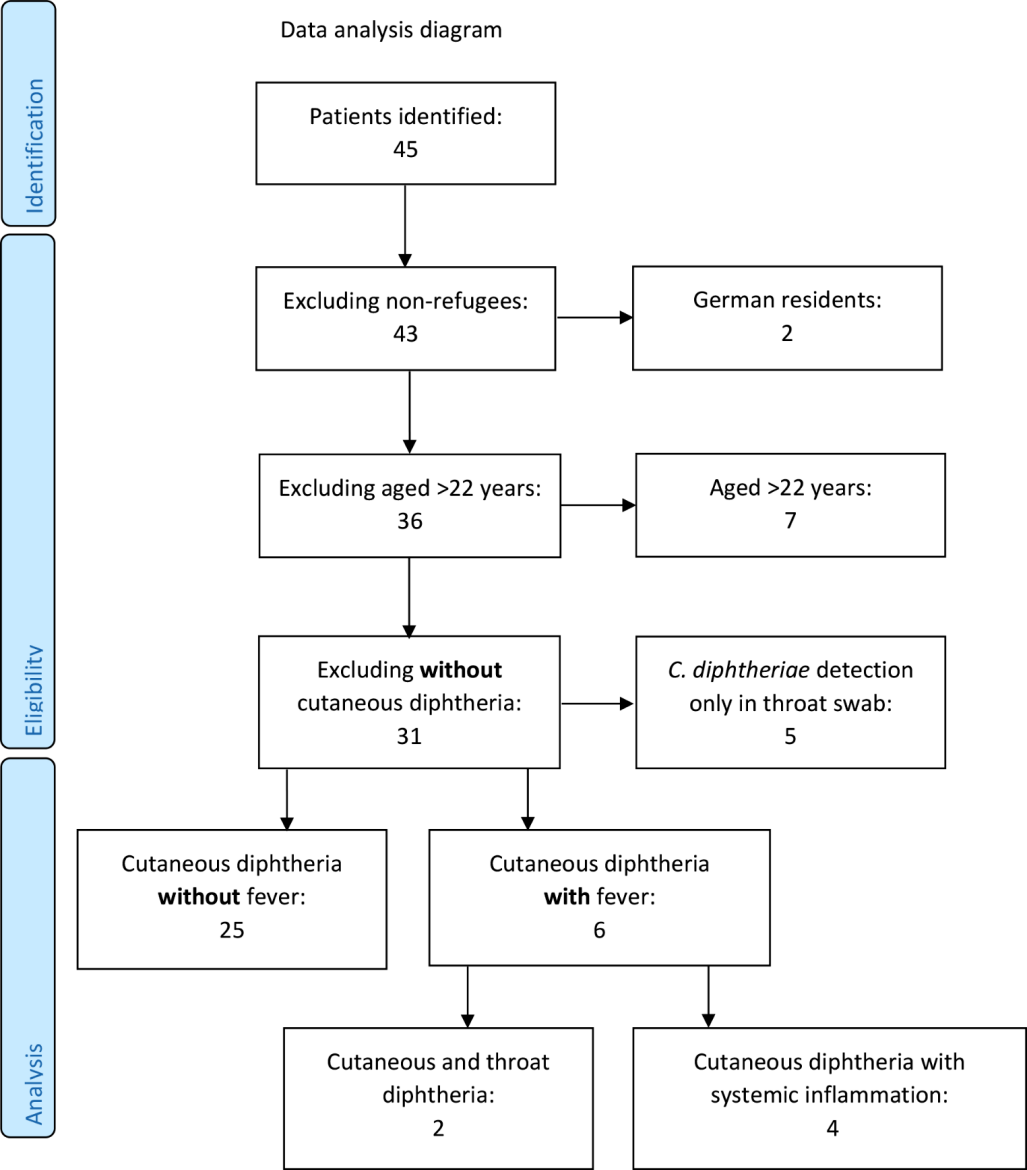
STROBE diagram for data analysis, adapted from [12]

A total of 45 patients were reported from nine centres. Two German residents without migration history and seven patients aged 22 years and older were excluded from further evaluation. Selection criteria were based on recent migration history, including refugees, asylum seekers and other migrants, due to their higher observed incidence and unique challenges such as environmental exposure during migration and rapid relocation. An additional five patients with microbiological detection of *C. diphtheriae* in throat swabs and without signs of infection were excluded, leaving a total of 31 adolescents with cutaneous diphtheria or *C. diphtheria*e-related wound infections and a recent migration history. Patients were divided into afebrile and febrile groups and compared for pharyngeal diphtheria and systemic inflammation. Systemic inflammation was defined by fever. A STROBE [12] diagram is shown in Fig. 1. This survey and the retrospective analysis were approved by the ethics committee of the University Medical Centre Freiburg (CUDAAR, ID 22-1493-S1-retro).

## Results

### Patient epidemiology and clinical presentation

All 31 patients were aged between 14 and 20 years at first presentation, with a mean age of 17 years. Most of them were from Afghanistan (total number 23; 74%), and Iraq (n 1; 3%), Syria (n 1; 3%) and Ukraine (n 1; 3%) were the other reported home countries. For five patients, no country of birth was reported. Twenty-one refugees were asked about their route of escape and all of them came via the Balkan route. Skin lesions commonly affected extremities, most frequently the feet (n 15; 48%), and the lower leg (n 13; 42%) followed by the hands (n 5; 16%). Other localizations included forearms (n 2; 6%), upper arms (n 2, 6%), genitals (n 2; 6%), ankle joint (n 2; 3%) and thigh (n 1; 3%) (supplement, Table 1).

**Fig. 2:**
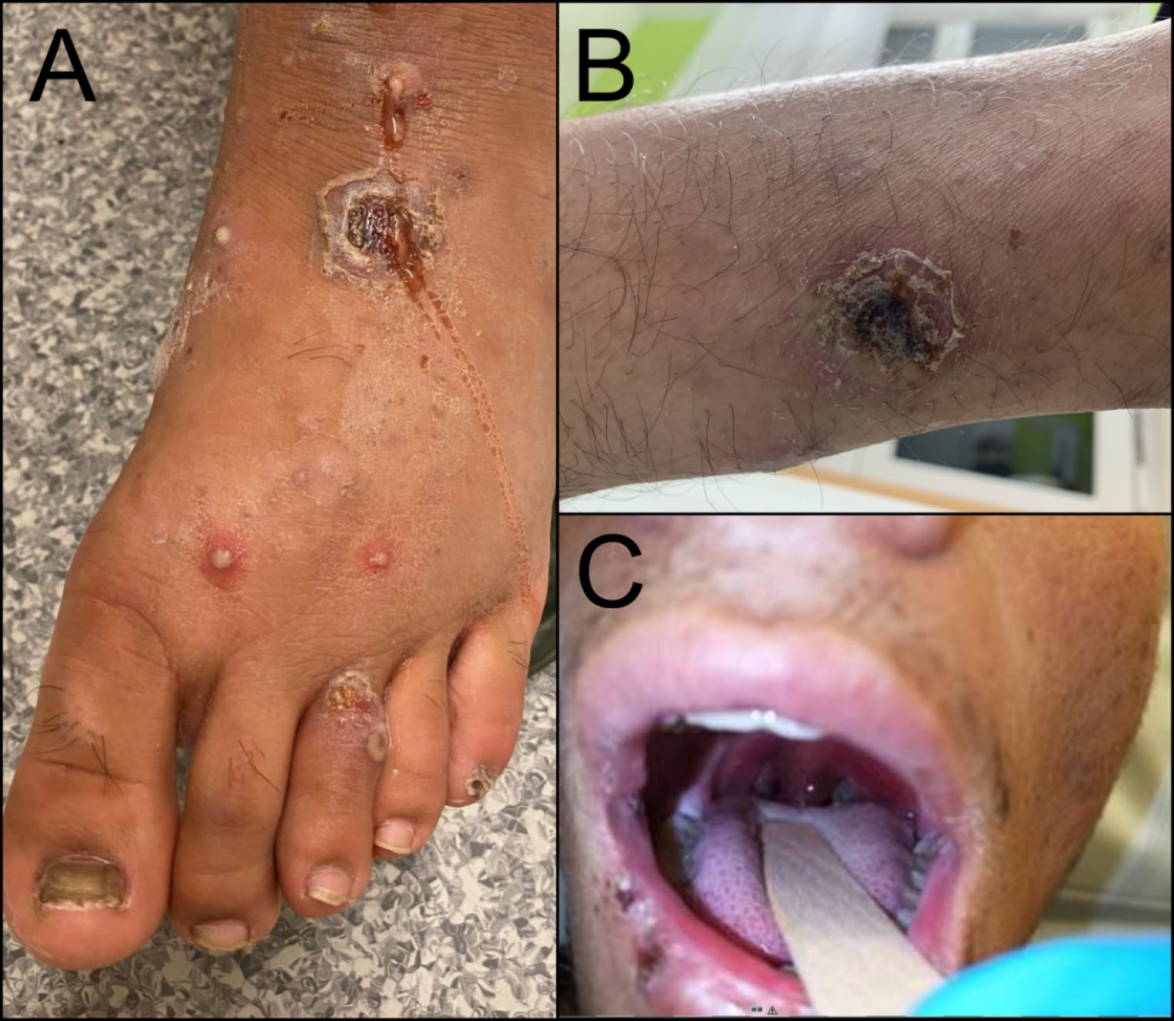
Clinical manifestation of cutaneous and pharyngeal diphtheria **A**: Multiple isolated erythematous pustules and purulent, partly erosive and ulcerated plaques with scratch excoriations and isolated haemorrhagic crusts on the foot of an adolescent from Afghanistan, who escaped via the Balkan route. Co-colonization with MRSA and *S. pyogenes* was detected, while a throat swab for *C. diphtheriae* was negative. Topical antiseptic and systemic antibiotic treatment with clarithromycin was carried out for 7 days, resulting in wound healing. **B**,** C**: Partly erosive and ulcerated plaque with scratch excoriations and isolated haemorrhagic crusts on the lower leg of a patient from Afghanistan. Treatment with Penicillin G was started. After 3 days, the patient presented again with fever and suspected pharyngeal diphtheria (C). Patient was admitted, antibiotic therapy was changed to doxycycline and the patient received Diphtheria Antitoxin Therapy (DAT). Antibiotics were administered for a total of 17 days and in the follow-up the throat swab showed *C. diphtheriae*-negative

Visually, the skin lesions appeared to be erosive with erythematous margins (Fig. 2A); some lesions showed papulo-pustules (Fig. 2A), while others appeared eschar-like with a central crust and desquamation (Fig. 2B).

Six of the 31 patients with chronic skin infection had fever at first presentation. One patient showed systemic signs of diphtheria toxin (DT)-related damage with clinical signs, i.e., atrio-ventricular block °III, which required implantation of a pacemaker. A second patient from this group showed the typical membranous lesions of diphtheria on the tonsils (Fig. 2C). Toxigenic *C. diphtheriae* was detected in throat swabs of both patients. Of the remaining four patients with fever, one had a positive blood culture with *C. diphtheriae*, and one had enlarged inguinal lymph nodes. The throat swabs for *C. diphtheriae* remained negative in these four patients. None of the 25 patients without fever at first presentation had signs of pharyngeal diphtheria or toxaemia and throat swabs taken in 19 of these 25 patients remained negative. The skin lesions of the 25 afebrile patients were comparable in location and appearance to those with fever.

### Microbiology results

All patients received a wound swab for pathogen detection and the majority also received a throat swab (n 25; 81%). The DT gene was detected by PCR in the majority of *C. diphtheriae* strains (n 25; 81%, supplemental Table 2). Additionally confirmation of toxin production with ELEK testing was reported for 4 patients. All febrile patients (n 6; 100%) and most afebrile patients (n 19; 76%) were toxin gene positive, with the toxin gene status unknown in four afebrile patients (supplement Table 2). Either *S. aureus* (n 6; 29%), *S. pyogenes* (n 2; 10%) or both (n 13; 62%) were commonly present in skin lesions. More than half of the isolated *S. aureus* strains were methicillin-resistant (n 10; 53%). *S. pyogenes* strains were partially resistant to cotrimoxazole (n 3; 15%) and macrolides (n 2; 10%). In this cohort, more than a quarter of *C. diphtheriae* were resistant to cotrimoxazole (n 10; 32%) and some were resistant to macrolides (n 2; 6%).

Apart from the different localisation of *C. diphtheriae*, there were no obvious differences in epidemiology, clinical manifestations, co-pathogens or antimicrobial resistance rates between patients with and without fever at initial presentation (data in supplementary Table 1 + 2).

### Treatment regimens

**Fig. 3:**
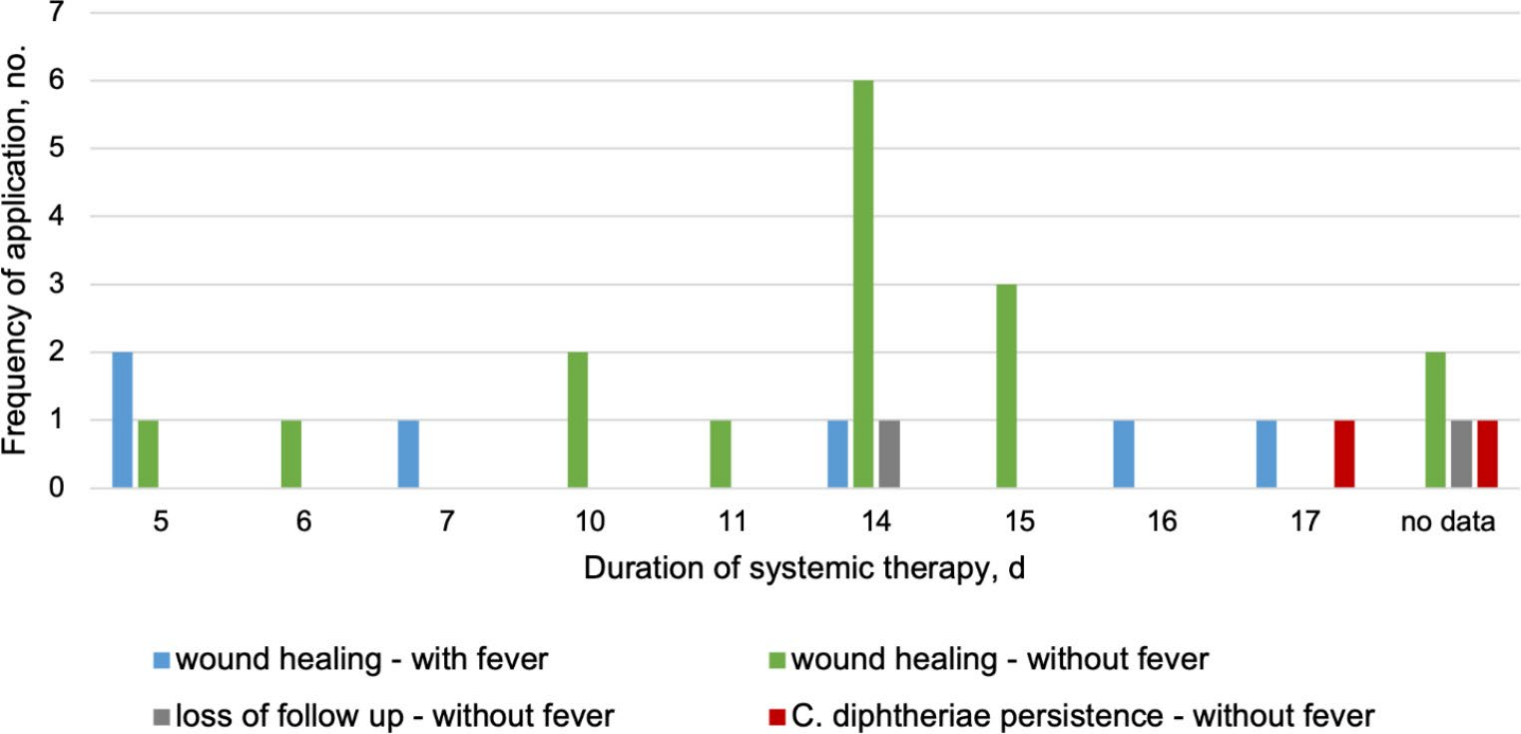
Treatment duration and outcomes stratified by fever vs. non-fever group. All patients with fever had resolution of skin wounds, with a broad distribution of treatment duration from 5–7 days to 14–17 days (light blue). Of the non-feverish patients, only two of 25 were lost to follow-up (grey) and two patients were persistently skin-colonized with *C. diphtheriae* (red). The remaining 16 patients showed resolution of skin infection (green) with variable duration from 5–8 days, but mostly 10–15 days

Topical treatment, most commonly with antiseptics, was given to most patients (n 23; 74%) independent of fever at initial presentation. Systemic antibiotic treatment was given to 26 (84%) patients. The five patients who did not receive systemic antibiotics were not successfully contacted after testing positive for *C. diphtheriae* in the wound swab for reassessment and, if necessary, modification of the therapeutic approach. One of these patients received a throat swab, which was negative. We definded treatment success as microbiologically confirmed eradication of *C. diphtheriae* or clinical wound healing, and treatment failure as persistence of the wound or continued detection of *C. diphtheriae*. Assessment was made at the end of treatment. For patients without fever, amoxicillin/clavulanic acid was the most frequently chosen systemic antibiotic (n 7; 35%). However, eradication of *C. diphtheriae* failed in two of these patients, although no aminopenicillin/BLI resistance was reported for the *C. diphtheriae* strains. Clindamycin was also commonly prescribed (n 5; 25%) and resulted in wound healing in all patients. Macrolides (n 5; 25%) and penicillin V (n 2; 10%) also resulted in favourable outcomes in patients without fever at initial presentation. Treatment duration in patients without fever was mostly 10 or 14 days, but shorter regimens of 5–8 days were equally successful (Fig. 3). Only two of the 25 patients (8%) were lost to follow-up.

All patients with fever at initial presentation received antibiotic therapy. The two patients with pharyngeal diphtheria had negative throat swabs after treatment, and their skin wounds were clinically resolved. The four febrile patients with negative *C. diphtheriae* throat swabs had either negative wound swabs (n 2) or clinically healed wounds (n 2); no further throat swabs were taken. The two patients with systemic signs of DT and pharyngeal diphtheria received penicillin G and diphtheria antitoxin as recommended by WHO until February 2024, and one patient was switched to doxycycline during treatment. In both cases, the duration of treatment was extended to 16 and 17 days, respectively. The four patients with fever and systemic inflammation received macrolides (n 2; 34%), penicillin G (n 1; 17%) or amoxicillin/clavulanic acid (n 1; 17%). In these patients, shorter antibiotic courses of 5 to 7 days showed a favourable outcome, with only one patient receiving a longer course of 14 days (Fig. 3).

To prevent spread of *C. diphtheriae*, some patients were kept in isolation. All febrile patients, including the two patients with detection of *C. diphtheriae* in pharyngeal swabs, were isolated (n 6; 100%), whereas only a minority of the non-febrile patients were isolated (n 11; 44%) (supplements, Table 3).

## Discussion

Chronic skin infections affect about 8–14% of adolescent and adult refugees seeking medical care [13–16]. Diagnosis and treatment can be challenging as many factors, such as low vaccination coverage, external environmental influences during escape or overcrowded asylum centres lead to a higher prevalence of infections that are rare in Europe. A striking increase in *C. diphtheriae* infections among refugees in Europe was observed starting in summer 2022 [7–9]. During the study period, 205 diphtheria cases were reported to the German National Health Authority. Thirty-six out of these were included in our study on adolescents. We carefully analysed the management in these CD cases with the aim to develop a treatment algorithm for refugees presenting with chronic wound infections at risk of *C. diphtheriae* infections.

In line with previous reports from us and others [7–9], escape via the Balkan route was overrepresented in refugees with CD in Germany. Chronic skin wounds were polymicrobial (*S. pyogenes*,* S. aureus*) in most cases, and among *S. aureus* isolates over 50% were methicillin-resistant (MRSA). This is consistent with previous reports [8, 17]. Our findings underscore that in refugees presenting with chronic skin wounds *C. diphtheriae* should be considered as a differential diagnosis and included in the diagnosis and management plan. In this cohort of adolescents, fever was a simple and quite specific parameter for distinguishing between severe cases of *C. diphtheriae* with potential for DT-related complications and cases with exclusive CD.

### Treatment for afebrile CD patients

WHO recommends macrolides and penicillin G for *C. diphtheriae* [18, 19], which is commonly sensitive to a range of antibiotics [20]. Surprisingly, in our cohort, amoxicillin/clavulanic acid (n 7) was most commonly used, but failed to eradicate *C. diphtheriae* in two of the seven patients. In contrast, clindamycin (n 5) showed a favourable outcome in all patients. Based on this observation and since most cases of cutaneous diphtheria were co-infections with other pathogens, choosing antibiotics that cover next to *C. diphtheriae* other common skin pathogens seems advisable. MRSA was a common co-pathogen that should be considered when selecting a systemic antibiotic. This limits the clinically rational treatment options to clindamycin, cotrimoxazole, doxycycline and macrolides. Yet, macrolide resistance is common in MRSA [21]. Additionally, macrolide resistance was found in two of the 31 (6%) *C. diphtheriae* isolates in our cohort. *C. diphtheriae* was resistant to cotrimoxazole in 32% of the isolates in our cohort, leaving clindamycin and doxycycline as preferable treatment options. As there are some concerns with the use of doxycycline (phototoxicity, pause after calcium-containing products), which could be problematic under the circumstances, clindamycin seems preferable. However, in the ongoing diphtheria European outbreak among refugees, one (ST-377) of the major outbreak clusters showed macrolide and clindamycin resistance [7, 22]. With respect to the findings in additional European countries [23], antibiotic resistance testing should be performed for every *C. diphtheriae* isolate. When broadening the perspective for cutaneous diphtheria-like illnesses, infections with toxigenic strains of the zoonotic pathogen *C. ulcerans* must be considered. *C. ulcerans* infections are usually acquired via animal contact (e.g., cats and dogs, but also via a wide variety of animals) and are often resistant to clindamycin. [24] While an increasing number of *C. ulcerans* infections has been reported in local pet owners [25], no cases of *C. ulcerans* have been reported in refugees in the literature yet [24–26], making *C. ulcerans* an unusual differential diagnosis.

Focusing on the European diphtheria outbreak among refugees, the following approach can be extrapolated from our data.

In refugees with chronic skin wounds without fever and in good general condition, we suggest performing wound and throat swabs for pathogen identification and antimicrobial resistance testing. Since *Corynebacteria spp.* are often summarized as “skin flora”, the microbiological laboratory should be informed about the clinical circumstances and the need to test for *C. diphtheriae*. Topical antiseptic therapy and oral antibiotic therapy with clindamycin should be instituted. Regarding length of therapy, most patients in our cohort received 10 to 14 days of systemic antibiotics, however, shorter courses of 5 to 8 days were also associated with good outcomes. Therefore, we suggest that an initial treatment duration of 7 days might be justified. This should be followed by clinical monitoring and microbiological clearance. A longer treatment course or a change of the antibiotic agent may be necessary depending on the clinical course or if microbial clearance has not been achieved. Diphtheria antitoxin should not be administered in these cases, both from a clinical point of view for the patient (high risk of anaphylaxis) and in view of the general shortage of diphtheria antitoxin stocks [2].

In all patients a thorough medical history should be taken, including duration of the lesion, route of escape and vaccination status, as well as a detailed clinical examination including a throat examination.

### Isolation recommendations

Less than half of the afebrile patients in our cohort were isolated to prevent transmission. Although preventive isolation measures are recommended for *C. diphtheriae* infected individuals [10, 27], difficulties in implementing isolation, such as overcrowding and lack of resources, may explain this. Isolated skin colonization, as in all our afebrile patients, carry a low risk of transmission if the wounds are appropriately covered. In the current outbreak, whole genome sequence analysis of 363 European cases of diphtheria infection identified three major clusters, yet transmission likely occurred outside Europe [22, 23, 28]. The phylogenetic analysis of 42 cases in Germany in 2022 yielded similar results, since no secondary cases could be identified [7]. It seems noteworthy that diphtheria vaccination coverage is high in Germany (96.9%, according to Robert Koch Institute) and across Europe (97%, according to WHO) [29, 30]. Balancing risks and resources, it seems justified not to isolate patients suspected of having isolated cutaneous diphtheria if adequate hygienic measures, such as covering wounds, the avoidance of close direct or indirect contact to the wound and fomites are in place. A recommendation to wear a mask until microbiology results are available is a resource-saving alternative. This approach could be implemented and adapted in conjunction with local health authorities. On the other hand, patients with suspected pharyngeal diphtheria, fever and poor general condition should definitely be isolated. For determination of the duration of isolation, we recommend microbiological eradication control after 5 days.

### Treatment for febrile CD patients

**Fig. 4:**
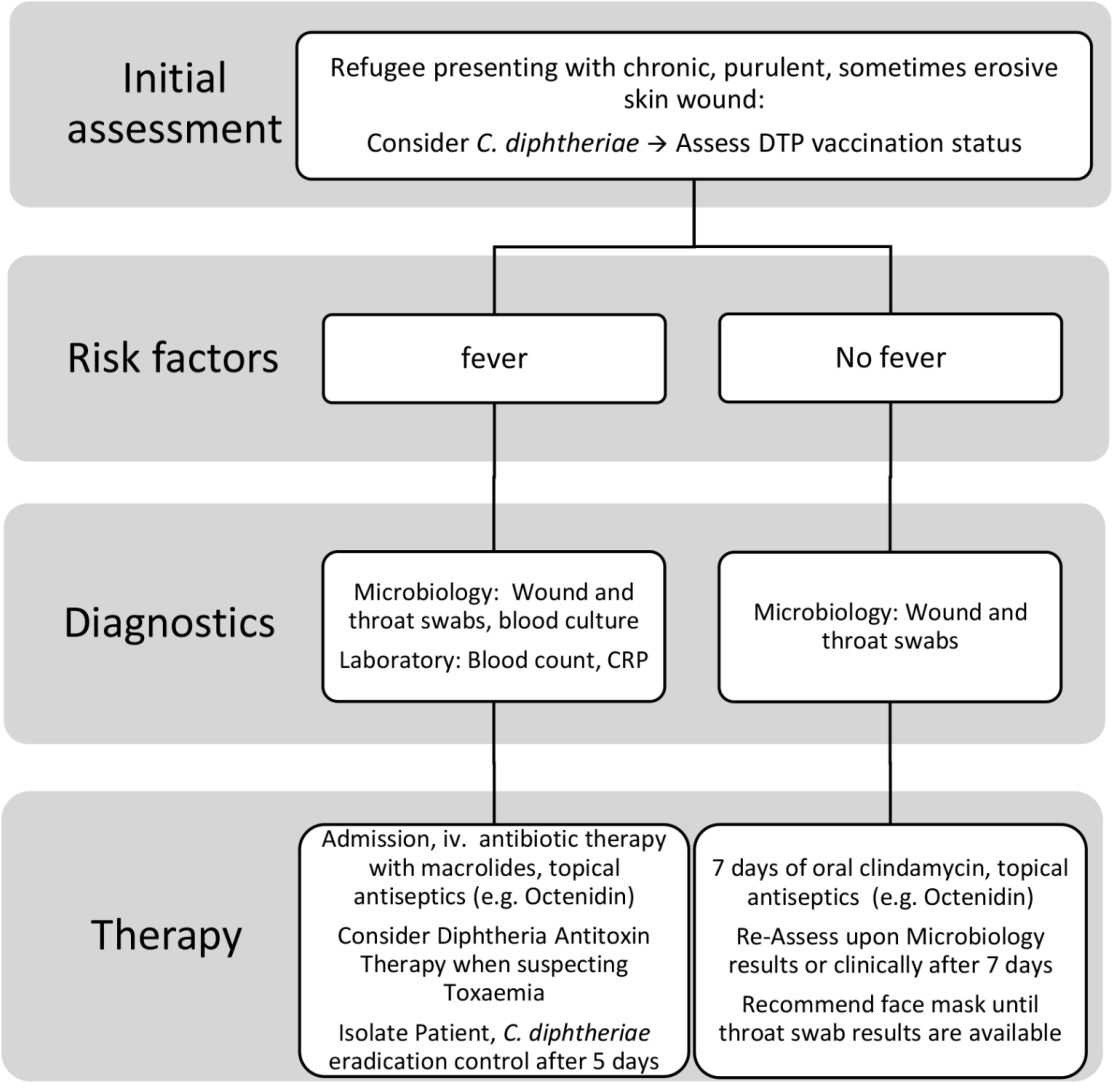
Proposed algorithm for treatment and diagnostics for refugees with chronic, purulent (erosive) skin wounds. DTP: Diphtheria, Tetanus, Pertussis vaccine; iv.: intravenous

Patients with cutaneous lesions and fever on initial presentation should be thoroughly examined and hospital admission must be considered. In the reported patients, a variety of antibiotic classes was employed, including penicillin G, amoxicillin/clavulanic acid, azithromycin, clarithromycin, doxycycline and combinations of these. Two patients with systemic diphtheria toxin-related symptoms also received antitoxin. In addition to a wound and throat swab, blood cultures must be taken, and if there are clinical signs of pharyngeal diphtheria or toxaemia, diphtheria antitoxin treatment (DAT) and antibiotic treatment according to WHO recommendations, i.e., macrolides or high-dose penicillin G, should be administered before results are available [11]. For febrile patients without signs of pharyngeal diphtheria, close clinical monitoring, thorough sampling and intravenous antibiotic therapy, preferably with clindamycin, may be sufficient in certain settings with suspected polymicrobial wounds and known clindamycin-sensitivity for *C. diphtheriae* in an outbreak setting. Our recommendation derived from this study and analysis of literature is summarized in Fig. 4.

This is to our knowledge the largest, detailed report on antimicrobial treatment for CD in central Europe. Although we were unable to obtain complete information on all points for all patients, it is noteworthy that only two patients in the afebrile group were missing data on treatment outcome. Due to the retrospective design only those individuals in whom *C. diphtheriae* was isolated from swabs could be included. There is a risk that cases may have been missed as *Corynebacteria spp*. may have been hidden under ‘skin flora’ or masked by faster growing bacteria, especially in mixed cultures. Furthermore, our report lacks information on the vaccination status of the patients, a common problem in refugee management, as in most cases vaccination records are not available. As vaccination coverage in countries of origin is sometimes low or incomplete [29], vaccinations should be given generously to all patients and to close contacts in conjunction with ECDC and national guidelines [10, 31].

## Conclusion

Our study provides insight into real-world treatment regimens in 31 adolescents with CD in Germany with a recent migration background. Cutaneous infections with *C. diphtheriae* were often polymicrobial with a high rate of MRSA and *S. pyogenes*. In all refugees with chronic skin wounds, a thorough medical history should be taken, including duration of the lesion, route of escape and vaccination status. Additionally a detailed clinical examination including a throat examination and wound and throat swabs, specifically investigating presence of *C. diphtheriae*, should be performed. In afebrile patients without signs of systemic DT or pharyngitis, an oral, seven-day course of clindamycin, accompanied by topical antiseptic treatment, seems to be a safe and rational treatment for cutaneous diphtheria. Patients presenting with fever require additional investigations, including blood cultures and benefit from hospital admission and close clinical monitoring. Upon signs of systemic DT, administration of DAT and preferably macrolides or high-dose penicillin G, maybe as part of a combination therapy, should be instituted. Vaccinations against diphtheria toxin should administered to all refugees in agreement with national guidelines.

## Electronic supplementary material

Below is the link to the electronic supplementary material.

Supplementary Material 1

## Data Availability

No datasets were generated or analysed during the current study.

## Declarations

### Use of artificial intelligence tools

No artificial intelligence was used in generating the research data or the manuscript.
