# Supplementary material for: Management and outcome of cutaneous diphtheria in adolescent refugees in Germany, June 2022 – October 2023
Source: Infection. 2024 Aug 27;53(1):329–37. doi: 10.1007/s15010-024-02374-y (PMC11825593; doi:10.1007/s15010-024-02374-y)
Supplement: Supplementary file 1 — Supplementary Material 1 [file 15010_2024_2374_MOESM1_ESM.docx]

**Supplements:**

| Demographics and clinical manifestations of 31 adolescent refugees with *Corynebacterium diphtheriae* in polymicrobial skin infections, from Germany, 01.06.2022 – 30.09.2023 | | | |
| --- | --- | --- | --- |
|  | No. (%) | | |
| Study population | Without fever  25 (81%) | With fever  6 (19%) | All patients  31 |
| Mean age at first assessment (range), y | 17 (14-19) | 17 (14-20) | 17 (14-20) |
| Country of birth |  |  |  |
| Afghanistan | 17 (68%) | 6 (100%) | 23 (74%) |
| Iraq | 1 (4%) | 0 (0%) | 1 (3%) |
| Syria | 1 (4%) | 0 (0%) | 1 (3%) |
| Unknown | 5 (20%) | 0 (0%) | 5 (16%) |
| Ukraine | 1 (4%) | 0 (0%) | 1 (3%) |
| Route of escape |  |  |  |
| Asked about route | 17 (68%) | 4 (67%) | 20 (65%) |
| Answered Balkan | 17 (100%) | 4 (100%) | 20 (100%) |
| clinical manifestation |  |  |  |
| Feet | 11 (44%) | 4 (67%) | 15 (48%) |
| Lower legs | 10 (40%) | 3 (50%) | 13 (42%) |
| Hands | 3 (12%) | 2 (33%) | 5 (16%) |
| Forearm | 2 (8%) | 0 (0%) | 2 (6%) |
| Upper arm | 2 (8%) | 0 (0%) | 2 (6%) |
| Genital | 2 (8%) | 0 (0%) | 2 (6%) |
| Ankle joint | 1 (4%) | 1 (17%) | 2 (6%) |
| Thigh | 1 (4%) | 0 (0%) | 1 (3%) |

**Table S1:** Demographics and clinical manifestations of 31 adolescent refugees with *Corynebacterium diphtheriae* in polymicrobial skin infections

| Microbiology test results for 31 adolescent refugees with *Corynebacterium diphtheriae* in polymicrobial skin infections, Germany, 01.06.2022 – 30.09.2023 | | | |
| --- | --- | --- | --- |
|  | No. (%) | | |
| Microbiology results | Without fever | With fever | All patients |
| Any skin wound with *C. diphtheriae* | 25 | 6 | 31 |
| Toxin gene *+* *C. diphtheriae* | 19 (76%) | 6 (100%) | 25 (81%) |
| Toxin gene *-* *C. diphtheriae* | 2 (8%) | 0 (0%) | 2 (6%) |
| Toxin status unknown | 4 (16%) | 0 (0%) | 4 (13%) |
| Any throat swab with toxigenic *C. diphtheriae* (% of all patients with *C. diphtheriae^pos.^* skin wound) | 0 (0%) | 2 (33%) | 2 (6%) |
| Co-colonization of skin infections (% of all skin wounds) | 21 (84%) | 5 (83%) | 26 (84%) |
| *C. diphtheriae, Staphylococcus. aureus, Streptococcus pyogenes* | 13 (62%) | 3 (60%) | 16 (62%) |
| *C. diphtheriae, S. pyogenes,* no *S. aureus* | 2 (10%) | 2 (40%) | 4 (15%) |
| *C. diphtheriae, S. aureus,* no *S. pyogenes* | 6 (29%) | 0 (0%) | 6 (23%) |
| Antimicrobial resistance |  |  |  |
| Total *S. aureus* isolates | 19 | 3 | 22 |
| MRSA (% of all detected *S. aureus*) | 10 (53%) | 3 (100%) | 13 (59%) |
| Total *S. pyogenes* isolates | 15 | 5 | 20 |
| Resistance to cotrimoxazole | 3 (20%) | 0 (0%) | 3 (15%) |
| Resistance to macrolides | 1 (7%) | 1 (20%) | 2 (10%) |
| Total *C. diphtheriae* isolates | 25 | 6 | 31 |
| Resistance to cotrimoxazole | 7 (28%) | 3 (50%) | 10 (32%) |
| Resistance to macrolides | 1 (4%) | 1(17%) | 2 (6%) |

**Table S2:** Microbiology test results for 31 adolescent refugees with *Corynebacterium diphtheriae* in polymicrobial skin infections

| Treatment and Isolation for 31 refugees with *Corynebacterium diphtheriae* in polymicrobial skin infections, Germany, 01.06.2022 – 30.09.2023 | | | |
| --- | --- | --- | --- |
|  | No. (%) | | |
| Treatment | Without fever 25 | With fever 6 | All patients  31 |
| topical therapy | 18 (72%) | 5 (83%) | 23 (74%) |
| topical antiseptic | 15 (83%) | 5 (100%) | 20 (87%) |
| topical antibiotic | 3 (17%) | 0 (0%) | 3 (13%) |
| Systemic therapy | 20 (80%) | 6 (100%) | 26 (84%) |
| Amoxicillin/Clavulanic acid | 7 (35%) | 1 (17%) | 8 (31%) |
| Clindamycin | 5 (25%) | 0 (0%) | 5 (19%) |
| Azithromycin | 3 (15%) | 1 (17%) | 4 (15%) |
| Clarithromycin | 2 (10%) | 1 (17%) | 3 (12%) |
| Penicillin G | 0 (0%) | 2 (33%) | 2 (8%) |
| Penicillin V | 2 (10%) | 0 (0%) | 2 (8%) |
| Ampicillin/Sulbactam | 1 (5%) | 0 (0%) | 1 (4%) |
| Doxycycline | 0 (0%) | 1 (17%) | 1 (4%) |
| Isolation of patient | 11 (44%) | 6 (100%) | 17 (55%) |

**Table S3:** Treatment for 31 refugees with *Corynebacterium diphtheriae* in polymicrobial skin infections
